# Assessment of brain functional connectome alternations and correlation with depression and anxiety in major depressive disorders

**DOI:** 10.7717/peerj.3147

**Published:** 2017-11-22

**Authors:** Vincent Chin-Hung Chen, Chao-Yu Shen, Sophie Hsin-Yi Liang, Zhen-Hui Li, Ming-Hong Hsieh, Yeu-Sheng Tyan, Mong-Liang Lu, Yena Lee, Roger S. McIntyre, Jun-Cheng Weng

**Affiliations:** 1 School of Medicine, Chang Gung University, Taoyuan, Taiwan; 2 Current affiliation: Department of Psychiatry, Chang Gung Memorial Hospital, Chiayi, Taiwan; 3 Department of Medical Imaging and Radiological Sciences, Chung Shan Medical University, Taichung, Taiwan; 4 Department of Medical Imaging, Chung Shan Medical University Hospital, Taichung, Taiwan; 5 Section of Child Psychiatry, Department of Psychiatry, Chang Gung Memorial Hospital at Taoyuan, Taoyuan, Taiwan; 6 Department of Psychiatry, Chung Shan Medical University and Hospital, Taichung, Taiwan; 7 Department of Psychiatry, Wan Fang Hospital & School of Medicine, College of Medicine, Taipei Medical University, Taipei, Taiwan; 8 Mood Disorder Psychopharmacology Unit, University Health Network, Department of Psychiatry, University of Toronto, Toronto, ON, Canada; 9 Institute of Medical Science, University of Toronto, Toronto, ON, Canada; 10 Departments of Psychiatry and Pharmacology, University of Toronto, Toronto, ON, Canada; 11 Current affiliation: Department of Medical Imaging and Radiological Sciences, Chang Gung University, Taoyuan, Taiwan

**Keywords:** Functional connectome, Graph theoretical analysis, Resting-state functional magnetic resonance imaging, Major depressive disorder

## Abstract

Major depressive disorder (MDD) is highly prevalent, recurrent, and associated with functional impairment, morbidity, and mortality. Herein, we aimed to identify disruptions in functional connectomics among subjects with MDD by using resting-state functional magnetic resonance imaging (rs-fMRI). Sixteen subjects with MDD and thirty health controls completed resting-state fMRI scans and clinical assessments (e.g., Hamilton Depression Rating Scale (HAMD) and Hospital Anxiety and Depression Scale (HADS)). We found higher amplitude of low frequency fluctuations (ALFF) bilaterally in the hippocampus and amygdala among MDD subjects when compared to healthy controls. Using graph theoretical analysis, we found decreased clustering coefficient, local efficiency, and transitivity in the MDD patients. Our findings suggest a potential biomarker for differentiating individuals with MDD from individuals without MDD.

## Introduction

Major depressive disorder (MDD) is highly prevalent, recurrent, and associated with significant functional impairment and morbidity (Richards, 2011). For example, MDD is associated with significant increases in all-cause, as well as suicide-related, mortality, reducing life expectancy by 7–11 years (Chesney, Goodwin & Fazel, 2014). The World Health Organization ranks MDD as a leading cause of disability and death worldwide (Murray et al., 2013). Neuropsychological evidence suggests that patients with MDD experience impairments in executive function, memory, and emotional processing (McIntyre et al., 2013). There is a pressing need to identify the neurobiological substrates of MDD to preempt individuals at elevated risk for MDD and to improve therapeutic outcomes.

Functional imaging techniques provide an opportunity to refine pathoetiological models of MDD. Numerous studies using fluorodeoxyglucose-positron emission tomography (FDG-PET) to measure cerebral glucose metabolism in MDD subjects and have reported significant decreased metabolism in prefrontal cortex, superior temporal gyrus, striatum, insula and cingulate cortex, whereas increased metabolism majorly in cerebellum, thalamus and limbic system, also in prefrontal cortex and cingulate cortex (Drevets, Bogers & Raichle, 2002; Kimbrell et al., 2002; Su et al., 2014). Recently, functional magnetic resonance imaging (fMRI), which is based on the blood oxygenation level-dependent (BOLD) effect to measure brain activity has been widening used for MDD approaches. fMRI measurements can be generally separated into task-based and task-free methods. Task-based fMRI studies are designed with stimulus protocols during MRI scan (for example, facial expression processing paradigm) and the brain activities in response to these stimuli are analyzed. The majority of task-based fMRI studies in MDD had been focused on evaluation of emotional dysfunction and had revealed that MDD subjects displayed dysfunction in multifocal brain areas, including prefrontal cortex, orbitofrontal cortex, sensorimotor cortex, limbic system (amygdala, parahippocampus), striatum, insula, fusiform, cingulate cortex and cerebellum compared to healthy controls (Delvecchio et al., 2012; Groenewold et al., 2013; Stuhrmann, Suslow & Dannlowski, 2011). However, task-based fMRI requires optimal task design which depends on study purpose and still lack consensus. Nervertheless, successful execution of the examination is highly dependent on the participant’s cooperation. In contrast, task-free fMRI, so-called resting- state fMRI (rs-fMRI), simplifies and facilitates the examination of brain activity during resting status of the participant with highly standardized and reproducible procedures and without any task-requirement (Biswal et al., 1995; Greicius et al., 2003; Raichle et al., 2001). Thus, rs-fMRI has therefore attracted attention by researchers interested in widening use in clinical applications. Amplitude of Low-Frequency Fluctuation (ALFF) is used to proxy the absolute intensity of spontaneous brain activity. These spontaneous low-frequency fluctuations have numerous similarities with fluctuations in neural metabolic, hemodynamic, and neurophysiological parameters (Zhang et al., 2014). Therefore, the ALFF during resting state is considered to be physiologically meaningful and reflective of spontaneous neural activity (Yue et al., 2015). A recent published meta-analysis of MDD, which investigated 32 rs-fMRI studies with different analysis methods, including ALFF, revealed alterations of spontaneous activity in multifocal brain areas in MDD subjects with decreased brain activity in superior frontal gyrus, superior and middle temporal gyrus, insula, precuneus, striatum and thalamus, whereas increased brain activity in prefrontal cortex, medial frontal cortex, cingulate cortex, parietal cortex, precuneus, hippocampus and cerebellum compared to healthy controls (Sundermann, Olde Lutke Beverborg & Pfleiderer, 2014). In addition previous studies have reported that individuals with MDD in remission continue to exhibit increased activation in the amygdala and decreased activation in the dorsolateral prefrontal cortex (Hooley et al., 2009). Several rs-fMRI studies have also reported on altered functional connectivity among individuals with MDD within and across multiple brain networks including, but not limited to, the default mode network (DMN) (Guo et al., 2013; Kaiser et al., 2015; Zhu et al., 2012). The foregoing results suggest a dysregulation of neural circuits inclusive of the prefrontal cortex as well as limbic structures (Romanczuk-Seiferth et al., 2014).

Postmortem studies have implicated alterations in the ventral areas of the prefrontal cortex (e.g., orbitofrontal cortex) in the pathogenesis of MDD (Aguilar et al., 2008; Monkul et al., 2007; Wagner et al., 2011). Convergent evidence suggests an association between suicidal behavior and functional connectivity. For example, Jollant et al. (2008) investigated neural reactivity following exposure to angry and happy versus neutral faces in currently euthymic men with a history of MDD, with or without suicidal behavior, when compared to male healthy control subjects. MDD and suicidal behavior were associated with activation of the right orbitofrontal cortex in response to negatively valenced emotional cues. They also reported associations between suicidal behavior and poor performance on measures of decision-making (Jollant et al., 2005), as well as between suicidal behavior and change in the activation of the orbitofrontal and occipital cortices, among individuals with MDD. A separate study reported lower activation of the left lateral orbitofrontal cortex among individuals with self-harming behavior when engaging in higher risk behavior (i.e., Iowa gambling task) when compared to lower risk behavior (Jollant et al., 2010). In addition, convergent evidence suggests that impairments in executive function (e.g., impulsivity, impaired risk assessment) are associated with suicidal ideation and behavior (Cooper et al., 2016; Lee et al., 2016). Studies using MRI have implicated structural alterations in several brain regions in the phenomenology of suicidal ideation and behavior (Beevers, 2005; Jollant et al., 2011; Van Heeringen, Bijttebier & Godfrin, 2011). For example, brain regions associated with suicidal behavior include, but are not limited to, the anterior cingulate cortex, right parietal lobe, and left temporal lobe among brain regions (Van Heeringen, Bijttebier & Godfrin, 2011). In addition, the regulation of negative emotional processing as well as impulse control (Beevers, 2005) are mediated by the medial, dorsomedial and dorsolateral prefrontal cortex. Furthermore, cognitive emotional biases (e.g., interpretation bias of a neutral event) are associated with abnormalities in the ventrolateral prefrontal cortex and limbic system, particularly the amygdala.

The human brain is structurally and functionally organized into complex networks, allowing for the segregation and integration of information processing. Brain networks have been shown to follow a specific topology known as “small-worldness” (Watts & Strogatz, 1998). A small-world network indicates a balance between network segregation and integration and is characterized by high local specialization and a high global integration between the brain regions. Graph theoretical analysis is one of the large-scale methods for understanding the functional connectome. Based on topology, graph theoretical analysis provides a novel insight to investigate the systematical alteration of the whole-brain functional organization and connection, including the small-worldness (*σ*), clustering coefficient (*C*), normalized clustering coefficient (γ), transitivity (*T*), characteristic path length (*L*), normalized characteristic path length (λ), local efficiency (*E*_local_), and global efficiency (*E*_global_) (Bullmore & Sporns, 2009; Rubinov & Sporns, 2010). *C*, *T* and *E*_local_ reflect functional segregation; *C* quantifies the extent of local interconnectivity in the network; *T* is similar to *C* but improves the disproportionately influenced by low degree nodes in *C* and *E*_local_ indicates how well the sub-graphs exchange information to each other. High scores on the three measures correspond to highly segregated neural processing. *L* and *E*_global_ reflect the functional integration; *L* measures the capability for information transfer between brain regions while *E*_global_ is a measure of the overall capacity for parallel information transfer and integrated processing. A lower *L* score or higher *E*_global_ indicates more rapid integration of specialized information from distributed brain regions. The γ and λ values are normalized relative to *C* and *L* of the 100 random networks. Small-worldness (σ) is calculated by dividing γ by λ (Bullmore & Sporns, 2009; Bullmore & Bassett, 2011; Hosseini, Hoeft & Kesler, 2012; Rubinov & Sporns, 2010). There have been several graph theoretical studies aiming to assess and compare the organization of structural and functional brain networks by using MRI in normal development, aging, organic and neuropsychiatric brain disorders, including MDD (Bullmore & Bassett, 2011; Gong & He, 2015; Lo, He & Lin, 2011; Ye et al., 2015; Zhang et al., 2011). Convergent evidence suggests that aberrant connectivity in brain networks subserving cognition, rather than abnormalities in a single brain region, is implicated in the pathophysiology of MDD.

The overarching aim of our analysis is to evaluate functional brain abnormalities in MDD patients when compared to healthy subjects using rs-fMRI. ALFF analyses will be conducted to detect local amplitude changes. Functional connectomic changes observed among MDD patients will be analyzed using the graph theoretical approach. The correlation between functional imaging indices and clinical assessments of depressive and anxiety symptomatology (i.e., Hamilton Depression Rating Scale, Hospital Anxiety and Depression Scale) will also be evaluated.

## Materials and Methods

### Study population

Participants were recruited from the Chung Shan Medical University Hospital in Taichung, Taiwan. The inclusion criteria for MDD subjects included a diagnosis of Diagnostic and Statistical Manual, Fourth Edition (DSM-IV)-defined MDD; a total score of >7 on the 17-item Hamilton Depression Rating Scale (HAMD); and receiving outpatient psychiatric care. Exclusion criteria for MDD subjects included history of schizophrenia or bipolar disorder, substance dependence during the past year (except for dependence on caffeine or nicotine), serious medical or neurological illness, current pregnancy or breastfeeding, and metallic implants or other contraindications to MRI. Exclusion criteria for healthy controls included: history of psychiatric, neurological illnesses or substance-use disorders; family history of major psychiatric or neurological illnesses; current prescription of any psychiatric or psychotropic medications; current pregnancy breastfeeding; and metallic implants or other MRI contraindications. The study was approved by the Institutional Review Board of Chung Shan Medical University Hospital (No. CS12209). All subjects participated in the study after providing informed consent.

### Clinical assessments

Participants were evaluated by a trained psychiatric nurse using the structured diagnostic interview of 5th edition Mini-International Neuropsychiatric Interview (MINI) (Sheehan et al., 1998), which has been validated in Taiwan (Chiang et al., 2007). The HAMD and Hospital Anxiety and Depression Scale (HADS) were used to assess the depression and anxiety symptom severity. Statistical analyses were conducted using statistical parametric mapping 8 (SPM8; Wellcome Department of Cognitive Neurology, London, UK) software.

### MRI data acquisition

All images were acquired using a 1.5-Tesla MRI (Signa HDxt; GE Medical System, USA) with an 8-channel head coil. The participants were instructed to lie down, remain motionless, keep their eyes closed, relax, think of nothing in particular, and remain awake. The functional images were obtained using the echo planar image (EPI) sequence. The image parameters were as follows: TR/TE = 2000/30 ms, FOV = 250 × 250 mm^2^, matrix = 64 × 64, in-plane resolution = 3.9 × 3.9 mm^2^, slice thickness = 4 mm, number of repetitions = 300, and 33 axial slices aligned along the anterior commissure-posterior commissure (AC-PC) lines. The fMRI scanning time was 10 min.

### Functional image pre-processing

Preprocessing was conducted using SPM8. After slice-timing correction, we calculated the center of each image and realigned the data to the first volume for motion correction (if the result of six head motion parameters exceeded 1 mm translation or 1° rotation they were excluded from this study). All of the participants were fitted to the criteria and no one was excluded. Following motion correction, data were normalized to standard Montreal Neurological Institute (MNI) space with affine transform and resampled to isotropic 3-mm voxels. The data were then spatially smoothed using an 8-mm full width at half maximum (FWHM) Gaussian kernel for better signal-to-noise ratio gain. Nuisance regression was then performed using the six head motion parameters as covariates. Then, the whole brain, WM, and CSF masks were used to remove the physiological noise. Linear de-trending and band-pass temporal filtering were performed on the time series of each voxel to minimize the effects of low-frequency drifts and physiological signals by the Resting-State Data Analysis toolkit v1.8 (REST v1.8; Center for Cognition and Brain Disorders, Hangzhou Normal University, Zhejiang, China). Previous studies suggested that the frequencies with important physiological information were in the range of 0.01–0.08 Hz (Cordes et al., 2001; Raichle et al., 2001). However, some research suggests that complex functional networks may be observed in the range of 0.1–0.12 Hz (Baria et al., 2011). Therefore, we extended the frequency range from 0.01 to 0.12 Hz to mitigate the influence of low-frequency drift and high-frequency physiological noise.

### Amplitude of low-frequency fluctuations analysis (ALFF)

The ALFF was calculated in the frequency range of 0.01–0.12 Hz. The procedure for calculating ALFF is briefly described as follows: for a given voxel, the time series was first converted to the frequency domain using a Fast Fourier Transform. The square root of the power spectrum was computed, averaged and normalized across a predefined frequency interval, which was termed the ALFF at the given voxel (Yue et al., 2015). We then performed two-sample *t*-tests with false discovery rate (FDR) correction to assess the difference in ALFF between the MDD patients and healthy controls. To investigate the relationship between the ALFF and HAMD/HADS scores, we calculated the correlation between ALFF and the HAMD/HADS scores of all participants with multiple regression by SPM8. In addition, gender, age, and education years were used as the covariates. To view the results, we used T1-weighted MNI template for creating the underlying map.

### Graph theoretical analysis

In graph theoretical analysis, we first defined a set of nodes and edges. Using the functional connectivity toolbox (CONN; The Gabrieli Lab. McGovern Institute for Brain Research MIT, United States of America), the whole brain was divided into 90 regions of interest (ROIs) (45 per hemisphere) with an automated anatomical labeling (AAL) template, each of which was considered a node (Behzadi et al., 2007; Whitfield-Gabrieli & Nieto-Castanon, 2012). The brain functional connectivity between two nodes could be represented as an edge. The degree of a node is the number of edges connecting it to the rest of the network, which allows us to characterize the edges distribution of all nodes in the network (Bullmore & Bassett, 2011).

The resting-state functional image was registered to the T1-weighted image and then to MNI space. The transformation matrix from resting space to MNI space was calculated by the transformation matrices created in the above two register processing steps and was stored for later use. We spatially normalized the resting-state functional images to the AAL template in MNI native space, and the connectivity matrix was obtained after functional connectivity analysis. The functional connectivity matrix was acquired from the functional connectivity toolbox (CONN; Neuroimaging Informatics Tools and Resources Clearinghouse, NITRC). Finally, we used the connectivity matrix to perform a graph theoretical analysis.

Analyses of network properties were performed with Graph Analysis Toolbox (GAT; Stanford University School of Medicine, Stanford, CA, USA) (Hosseini, Hoeft & Kesler, 2012). Previous analysis produced a 90 × 90 association connectivity matrix for each individual. GAT extracted the regional mean time series of each of the 90 ROIs, and partial correlation was used to construct undirected weighted networks. Before statistical analyses, the density range in which a network comparison is meaningful needs to be identified (i.e., the density range in which the networks are not fragmented) (Hosseini, Hoeft & Kesler, 2012). After examining all of the networks, the minimum network density at which no individual network was fragmented was identified as 0.15. The maximum density of the network is determined by the percent of connections present using the most lenient threshold applied, which is 0.4. Next, the networks of the two groups were created at different correlation thresholds, ranging from 0.15 to 0.4, in 0.01 increments. To determine the statistically significance differences between groups in the network topology and regional network measurements, GAT was used to perform the two-sample *t*-test and non-parametric permutation test with 1,000 repetitions.

## Results

### Study participants

A total of 46 participants were recruited and enrolled in the study, including 16 MDD patients and 30 age- and sex-matched healthy controls (all of the participants were fitted using the criteria of six head motion parameters in the realignment process). The ages of the MDD subjects ranged from 24 to 52 years (mean 44.81 ± 2.2) and those of the control subjects ranged from 24 to 58 years (mean 45.03 ± 1.88). Other clinical and sociodemographic characteristics of study participants are reported in Table 1.

**Table 1:** Demographic and clinical characteristics.

	MDD	Healthy subjects	*P*-value
Age (years) ± SE	44.81 ± 2.2	45.03 ± 1.88	0.87
Range of age (years)	24–52	24–58	–
Gender (male/female)	(3/13)	(3/27)	–
HAMD score	9.31 ± 1.77	0.9 ± 1.9	<**0.001**
HADS (anxiety subscale) score	10.93 ± 1.42	2.58 ± 0.48	<**0.001**
HADS (depression subscale) score	10.31 ± 1.35	2.00 ± 0.36	<**0.001**

**Notes.**

SE Standard error of mean HAMD Hamilton Depression Rating Scale HADS Hospital Anxiety and Depression Scale

### Amplitude of low-frequency fluctuations analysis (ALFF)

**Figure 1:**
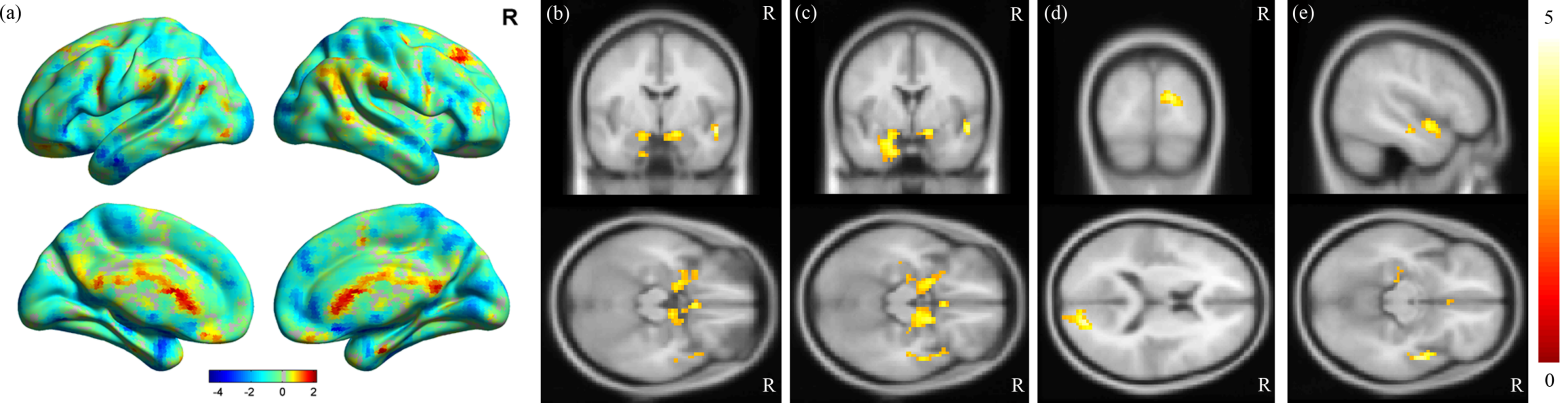
Results of ALFF analysis. (A) Surface view of ALFF analysis, where the color bar represents *t*-scores. Red represents higher ALFF in the control groups, and blue represents higher ALFF in the MDD groups. MDD brains showed increased ALFF in the (B) bilateral hippocampus, (C) bilateral amygdala, (D) calcarine and (E) superior temporal gyrus, with a corrected *p*-value of less than 0.01. The color bar represents *t*-scores.

**Figure 2:**
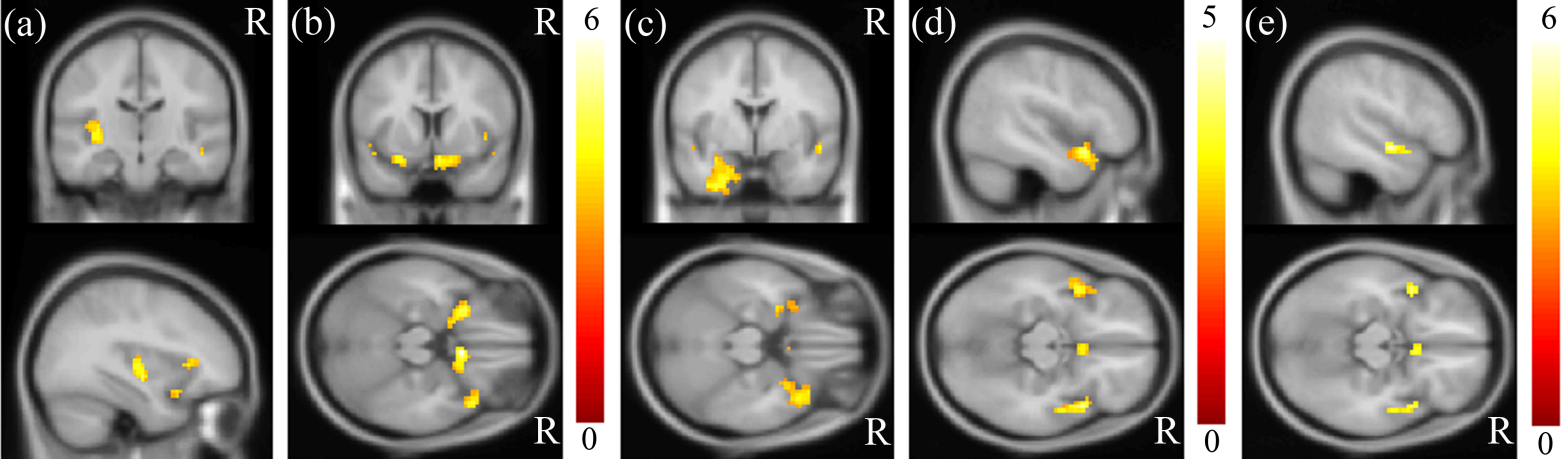
Results of correlation analysis between ALFF and HAMD/HADS scores. A highly positive correlation between ALFF and the HAMD total score was found in the (A) insula and (B) para-hippocampus (*p* < 0.01). A highly positive correlation between ALFF and the HADS anxiety subscale score was found in the (C) amygdala and (D) superior temporal gyrus (*p* < 0.01). A highly positive correlation between ALFF and the HADS depression subscale score was identified in the (E) superior temporal gyrus (*p* < 0.01). The color bar represents the *t*-scores.

MDD was associated with significantly increased ALFFs in the bilateral temporo-limbic regions, including the hippocampus, amygdala, superior temporal gyrus and also calcarine (*p* < 0.01) (Fig. 1). No between-group differences in decreased ALFFs were found. In the correlation analysis between ALFF and clinical scores, a highly positive correlation between the ALFF and HAMD total score was found in the insula and para-hippocampus (*p* < 0.01) (Figs. 2A and 2B), a robust positive correlation between ALFF and HADS anxiety subscale total score was found in the amygdala and temporal lobe (*p* < 0.01) (Figs. 2C and 2D) and a highly positive correlation between ALFF and the HADS depression subscale total score was identified in the temporal lobe (*p* < 0.01) (Fig. 2E).

### Graph theoretical analysis

**Figure 3:**
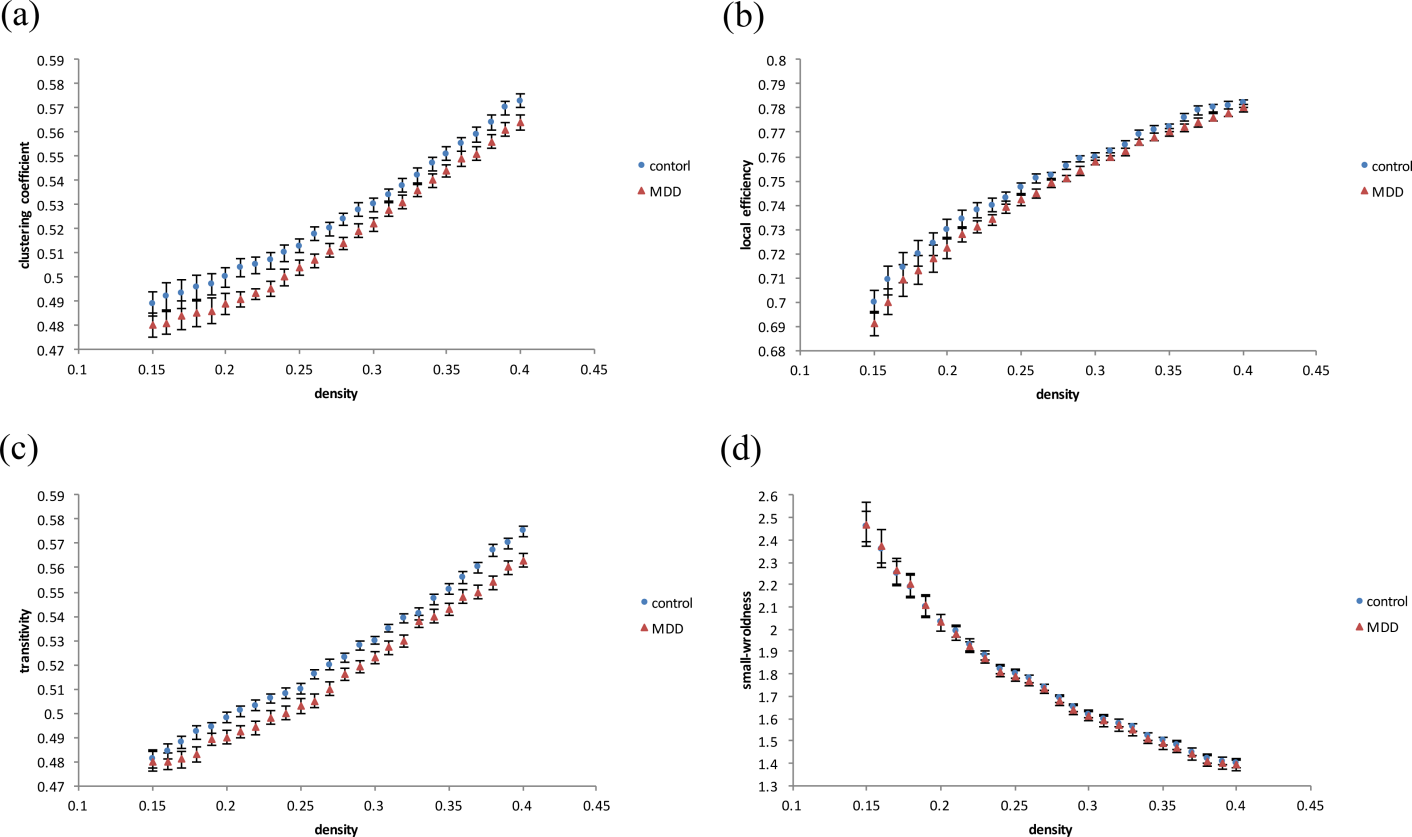
Results of degree distribution and topological measurements. The MDD group showed a significantly decreased (A) clustering coefficient, (B) local efficiency and (C) transitivity (*p* < 0.05). There was no significant difference in (D) small-worldness between the MDD and control patients.

In graph theoretical analysis, we used the GAT toolbox to calculate the functional connectivity matrices for both groups. When compared to healthy controls, MDD subjects had significantly decreased clustering coefficients (Fig. 3A), local efficiency (Fig. 3B), and transitivity (Fig. 3C) in whole-brain network topological organization (*p* < 0.05). There was no significant difference in global efficiency (including characteristic path lengths and normalized characteristic path lengths) or small-worldness (Fig. 3D). The density results represent the ratio of existing connections to all possible connections.

## Discussion

In this study, we used resting-state fMRI with ALFF and functional connectome approaches for MDD patients and healthy controls evaluation. In addition, we compared changes in brain functional connectivity and topology with clinical assessments of depressive and anxiety symptomatology. First, we found significant increased ALFF among MDD subjects bilaterally in temporo-limbic regions, including the hippocampus, amygdala, superior temporal gyrus, and calcarine. Second, there were statistically significant positive correlations between ALFF, HAMD and HADS total scores in bilateral temporo-limbic regions. Moreover, we found decreased functional clustering coefficient, local efficiency and transitivity among MDD subjects using graph theoretical analysis.

Biswal et al. (1995) first reported that ALFF in brain reflects the temporal changes in neural spontaneous activity. Several recent studies have used rs-fMRI with ALFF analysis to evaluate first-episode and treatment-free MDD patients and reported altered ALFF/ fractional ALFF and different ALFF frequency ranges in various brain regions, including frontal lobes, temporal lobes, parietal lobes and occipital lobes of the cerebrum, as well as anterior and posterior lobes of the cerebellum compared to healthy controls (Lai & Wu, 2015; Wang et al., 2012; Yue et al., 2015). The distributed pattern of brain regions with increased or decreased ALFF in MDD patients compared to healthy controls of the aforementioned studies, which enhanced that MDD is a complicated, variable, multiple brain areas involved disorder, especially in first-episode and treatment-free patients. In contrast, the MDD subjects enrolled in our study had been receiving regular psychiatric care, which may lead to the results that the ALFF alterations were only limited in certain areas, and consistently increased ALFF, majorly localized in bilateral temporo-limbic regions.

Changes in the temporo-limbic regions are a replicated finding in MDD populations. The temporal lobe is involved in processing received stimuli and subserves social cognition, memory, and emotional processing (Ramezani et al., 2014). The hippocampus and amygdala both belong to the limbic system and play important roles in long-term memory and emotional regulation (Phelps & LeDoux, 2005). A recent meta-analysis study reported a significant reduction in grey matter in limbic regions in untreated MDD patients (Zhao et al., 2014). Furthermore, in our recently published study, we found that MDD patients, including those receiving treatment, have decreased hippocampal and amygdalar volumes when compared to healthy controls; in addition, lower hippocampal and amygdalar volumes were significantly associated with greater HAMD and HADS total scores (Chen et al., 2016).

The most commonly used antidepressants among our MDD subjects were selective serotonin and noradrenergic reuptake inhibitors. A recently published systematic review reported that treatment response to antidepressants (selective serotonin/noradrenergic reuptake inhibitors) were associated with increased functional connectivity between limbic and frontal brain regions, which may be relevant to abnormal cognitive-emotional processing commonly observed in subjects with MDD (Dichter, Gibbs & Smoski, 2015). Therefore, increased ALFF in the temporo-limbic regions in our MDD subjects may be partly mediated by the effects of antidepressants. However, results from our graph theoretical analysis indicate that our MDD subjects exhibited decreased functional clustering coefficients, local efficiency and transitivity, suggesting poor function of local segregation, and indicating a shift toward randomization in their functional brain networks. These findings revealed that the local segregation of the resting network was influenced by MDD and disrupted networks may lead to impaired organization of brain networks for information transfer. Taken together, increased temporo-limbic ALFF, as well as decreased local functional segregation, may result in insufficient and disrupted functional connectivity between limbic and frontal brain regions, which may manifest as clinically significant depressive and anxiety symptoms.

The DMN is a large-scale brain network believed to support internally oriented and self-referential thought that contains specific brain regions, including medial prefrontal cortex, anterior cingulate cortex, posterior cingulate cortex, precuneus, dorsomedial thalamus and medial, lateral, and inferior parietal regions, which shows deactivation during certain task-related activity but shows activation during passive wakeful resting status (Greicius et al., 2003; Raichle et al., 2001). Previous rs-fMRI studies suggested that alteration of the DMN plays an important role in MDD patients (Guo et al., 2013; Kaiser et al., 2015; Ye et al., 2015; Zhu et al., 2012). However, our results showed no significant difference of the DMN between the MDD subjects and the healthy controls. We believed the majority cause of the inconsistency may result from the fact that our MDD subjects underwent regular psychiatric care. This argument could be supported by several recent studies which focused on treatment responses of MDD patients and suggested that medical treatment can effectively normalize alteration of the DMN in treatment sensitive MDD patients (Guo et al., 2012; Li et al., 2013; Liston et al., 2014; Posner et al., 2013).

There are several limitations that may limit interpretations of and inferences made from the results reported herein. First, the sample size used was relatively small, which may affect the generalizability of the results. Thus, future research should involve a larger sample size of depressive patients. Second, the cross-sectional design did not allow us to observe treatment effects in the MDD participants. Hence, longitudinal studies are needed to examine such effects.

## Conclusions

We used functional connectome analysis to investigate brain functional alternations in MDD patients. We found higher ALFF in the bilateral temporo-limbic regions in MDD patients when compared to healthy controls. In addition, a decrease in local functional segregation in the MDD patients was observed. Our results could be used to identify candidate imaging biomarkers for clinical diagnosis of mood disorders and, as well, our results aid in the understanding of the underlying pathophysiology of depression. Elucidation of these image markers will provide novel insights for further classification or evaluation of treatments for MDD.

## Additional Information and Declarations

### Data Availability

The following information was supplied regarding data availability: Weng, JC (2016): MDD_fMRI. figshare.
https://doi.org/10.6084/m9.figshare.4220040.v1.
